# *Ascaris* from Humans and Pigs Appear to Be Reproductively Isolated Species

**DOI:** 10.1371/journal.pntd.0004855

**Published:** 2016-09-01

**Authors:** Martin Jensen Søe, Christian M. O. Kapel, Peter Nejsum

**Affiliations:** 1 Department of Plant and Environmental Sciences, Faculty of Science, University of Copenhagen, Frederiksberg, Denmark; 2 Department of Veterinary Disease Biology, Faculty of Health and Medical Sciences, University of Copenhagen, Frederiksberg, Denmark; University of Melbourne, AUSTRALIA

## *Ascaris* Taxonomy Is Contentious

The taxonomic status of *Ascaris lumbricoides* and *A*. *suum* is contentious: shall they be considered a single species? Yes, as argued by Leles et al. [1], and most recently debated through opposing views by Alves et al. [2] and Betson et al. [3]. Most taxonomic literature refers to two species, and results from investigations of the nuclear DNA of *Ascaris* isolates of human and pig origin suggest that they are reproductively isolated and thereby fulfill the biological species definition. In our opinion, two species should therefore still be recognized until convincing evidence is presented to justify a reclassification.

And yes, a number of studies have compared *Ascaris* isolates from pigs and humans and have identified close similarities in mitochondrial [4–6] and single nuclear markers (such as internal transcribed spacer [ITS] [5,7] and 18S rRNA [8]), including shared genotypes and haplotypes. It has been clearly established that *Ascaris* can cross infect hosts experimentally and in natural settings, as reviewed by Nejsum et al. [9].

## Nuclear DNA Markers Segregate Worms According to Host and Geographical Origin

However, four studies have examined highly variable nuclear markers—that is, microsatellites and amplified fragment length polymorphisms (AFLP)—in worm isolates of human and pig origin from many different geographic locations and identified distinct segregation according to host and geographical origin:

Study 1: Criscione et al. [10] showed genetic differentiation among all tested populations using microsatellite markers. The dataset comprised 97 human isolates from China, Guatemala, and Nepal and 32 pig isolates from China and Guatemala, among which four hybrids were detected in humans. A single host shift between pigs and humans was not demonstrated, suggesting that host-associated populations emerged after geographical isolation.

Study 2: Zhou et al. [7] examined microsatellite markers in 137 human and 121 pig isolates from China and identified cross infections, which most often (19 of 20 cases) occurred by infection of humans with pig-type *Ascaris*, in addition to 20 identified hybrid cases, of which again most (19) were found in humans. Even though this study does not include global sampling, it suggests that there is restricted gene flow between human and pig *Ascaris* in China.

Study 3: The most comprehensive dataset was presented more recently by Betson et al. [4], in which 246 human and 164 pig isolates from Europe, Africa, and Asia were examined using microsatellite markers. In a sympatric area (Kabale district), two cases of cross infections were identified (one in each host) out of a total of 199 worms, whereas all worms from humans in Europe were identified as pig *Ascaris*. In general, worms segregated first by host and then by geographic location, with significant genetic differentiation between populations.

Study 4: Nejsum et al. [11] examined 71 human (Denmark, Bangladesh, Guatemala, and Nepal) and 64 pig isolates (Denmark, Guatemala, and Philippines) using AFLP, a whole-genome multilocus “fingerprinting” approach. *Ascaris* worms clearly segregated according to host origin; pig *Ascaris* were found in one cluster, whereas two clusters were observed for human *Ascaris* (Guatemala versus Nepal and Bangladesh). However, this study is restricted to a limited sample size (15) from a sympatric area (Guatemala) and the unrooted tree, as depicted in Figure 2, cannot be used to infer phylogeny.

## Hybrids Appear Unfertile, Indicating Reproductive Isolation

In these studies, the first two identified hybrids [7,10], and all four identified cross infections [4,7,10,11]. Importantly, they also demonstrated distinct assignments to the host origin. In Criscione et al. (2007), the Fst between humans and pigs in China and Guatemala were 0.186 and 0.302/0.307, respectively [10]. If we consider an island model with Fst = 1 / (4N_e_m + 1), a population of 1,000 worms (N) that has an effective population size of 100 (N_e_), and the above given Fst values, this suggests 0.01 and 0.006 migrants per generations in Guatemala and China, respectively, under these given assumptions. However, relatively high numbers of hybrids were detected in Guatemala (4%) [10] and China (7% and 7.8%) [7,10]. If we consider the hybrids as representatives of successful migrants between the two host populations, this would correspond to 40–78 migrants in a population of 1,000 worms. This suggests that these hybrids have very low fertility or are not fertile at all, as this level of migration would break down the genetic structure between the two populations. In relation to this, *Ascaris* from pigs in Uganda were genetically more closely related to *Ascaris* from pigs in Denmark than *Ascaris* from humans in Uganda [4]. In contrast, the Fst between humans and pigs in China were substantial lower (0.186) than between humans in China and Guatemala. However, it should be noted that the Fst is surprisingly even lower between pigs in Guatemala and humans in China (0.173). Nonetheless, these studies indicate that *Ascaris* from humans and pigs are reproductively isolated.

## Evolutionary Relationship Is Not Yet Fully Elucidated

The evolutionary relationship between *Ascaris* in humans and pigs is, however, not fully elucidated. In Betson et al. (2014), *Ascaris* populations were first separated by host and then by location, suggesting a single host shift followed by geographical separation [4]. In contrast, Criscione et al. found support for a model in which the ancestral *Ascaris* population was first geographically separated and then multiple host shifts occurred. Considering that gene flow is subsequently restricted, as the above evidence points to, we may have a situation of multiple species of *Ascaris* in humans and pigs across the globe, with possible implications for transmission and control. It should, however, be noted that there was low bootstrap support for the internal branches of the dendrograms in Criscione et al., which may reflect the lower-than-expected Fst between pigs in Guatemala and humans in China [10].

So, why do we still see shared haplotypes between worms from humans and pigs and observe a low phylogenetic resolution when using mitochondrial DNA (mtDNA) markers or single nuclear genes? Considering that one or more potential host shifts have occurred for *Ascaris*, this has most likely taken place during several pig domestication events that are estimated to have been initiated around 10,000 years ago [12]. In Betson et al. 2014, the p-distance between the haplotypes H01 and H64 is 0.052 [4]. In a mitochondrial genome of 14,000 bp, this corresponds to approximately 730 changes. Assuming a mutation rate equal to that of the related nematode *Caenorhabditis elegans* [13] (1.6 x 10^−7^ mutations per site per generation) would lead to an estimate of 325,000 generations since the divergence of these two haplotypes, predating pig domestication events. Thus, the presence of shared haplotypes most likely represents retention of ancestral polymorphisms, but we recognize that introgression (or a combination of the two) also may explain these observations.

Even though *Ascaris* in humans and pig appear reproductively isolated with no or very limited gene flow, the zoonotic properties of *A*. *suum* is evident from cross infection studies and transmission under natural settings. Interestingly, it appears that humans are more often infected with pig *Ascaris* or hybrids than vice versa, at least in China [4,7,10]. The reason for this may relate to differences in exposure and/or host suitability. In relation to this, a permanent transmission cycle of *A*. *suum* seems to have established among chimpanzees in Copenhagen Zoo [14], but whether *A*. *suum* in general is more likely to cross infect than *A*. *lumbricoides* needs to be elucidated. Nonetheless, there is a need to implement a “one health” approach in order to control *Ascaris* infections.

## Genome-Wide Population Studies Hold the Key

Fortunately, next-generation sequencing may provide whole genome sequences of multiple individuals, as exemplified for *Trichinella* [15] and *Schistosoma* [16] worms. Comparable approaches may be applied to *Ascaris* isolates from worldwide locations, with a focus on sympatric areas. This opens up for performing in-depth comparative genomic studies of *Ascaris* populations and thereby provides unprecedented insights into the dispersal and evolution of *Ascaris* as well as more definitive conclusions as to whether or not one, two, or even multiple species should be recognized. Such studies may also identify signatures in the genome associated with host adaptation and speciation, and in relation to this, it would be of particular interest to include worms that have been identified as hybrids and cross infections.
